# Regulation of Proximal Tubule Phosphate Transport: New Partners, New Pathways

**DOI:** 10.3390/cells15151369

**Published:** 2026-07-29

**Authors:** Eleanor Lederer, Syed Jalal Khundmiri

**Affiliations:** 1 Medical Services, VA North Texas Health Care Services, Dallas, TX 75216, USA; 2 Department of Medicine, University of Texas Southwestern Medical Center, Dallas, TX 75390, USA; 3 Charles and Jane Pak Center for Mineral Metabolism and Clinical Research, Dallas, TX 75390, USA; 4 Department of Medicine, University of Louisville School of Medicine, Louisville, KY 40202, USA; 5 Department of Biophysics and Physiology, Howard University, Washington, DC 20059, USA; syed.khundmiri@howard.edu

**Keywords:** phosphate homeostasis, phosphate transporter, cell signaling, PDZ domain

## Abstract

Disorders of phosphate homeostasis are common but difficult to treat. The major organ systems responsible for phosphate homeostasis are the intestine, bone, kidney, and parathyroid gland. Overall, kidney handling of phosphate is the major regulator of both serum phosphate levels and overall phosphate homeostasis. However, our ability to manipulate kidney phosphate handling has been very limited. Npt2a has been more thoroughly investigated than Npt2c, but recent studies identifying the role of Npt2c in human disease have uncovered key differences in the role of Npt2c in mice and humans. Recent studies have identified new proteins, such as TMEM174 and RGS14, that are involved in the regulation of the major sodium-dependent phosphate transporters in the kidney. Additionally, newer chemical agents to alter the function of these transporters have been developed. The purpose of this review is to highlight these new advances, place them in the context of our current knowledge of phosphate homeostasis, and explore potential new approaches to abnormalities of phosphate homeostasis. The recent discoveries suggest potential therapeutic targets for hyperphosphatemic conditions, such as chronic kidney disease and tumoral calcinosis, as well as for hypophosphatemic states, such as Fanconi syndrome, hypophosphatemic rickets, or transporter mutations.

## 1. Introduction

Phosphate is critical for health. It is ubiquitously present in virtually all biological processes as a component of mineralized tissue; a key element for energy storage molecules and genetic information; and a regulator of the structure and function of proteins, lipids, and other cell components [1]. Not surprisingly, chronic abnormalities in the regulation of serum phosphate result in disease. Hypophosphatemia and phosphate deficiency lead to decreased bone mineralization, rickets, and, if sufficiently severe, muscle weakness, rhabdomyolysis, seizures, decreased cardiac function, and respiratory failure [2,3]. Hyperphosphatemia leads to extraskeletal mineralization, including vascular and heart valve calcifications, calciphylaxis, and an increased risk of cardiovascular events [4,5,6,7,8,9].

## 2. Phosphate Homeostasis

Phosphate homeostasis is maintained through the integrated function of the gastrointestinal tract, kidneys, bone, and parathyroid glands. Dietary ingestion of phosphate far exceeds the daily requirements, resulting in an excess that is then excreted by the kidneys. The regulation of intestinal phosphate uptake is incompletely understood; however, the paracellular pathway for intestinal phosphate uptake predominates in humans, while transcellular transporter-mediated uptake is a minor component [10]. The regulators of paracellular intestinal phosphate uptake are not known, but the inhibitory effects of tenapanor—an oral inhibitor of the sodium hydrogen exchanger (NHE3)—suggest that pH may be a factor [11,12,13]. There are likely additional factors modulating paracellular phosphate transport, as a recent study demonstrated that the deletion of intestinal epithelial cell NHE3 resulted in enhanced phosphate uptake [14]. This predominance of the paracellular pathway ensures that daily absorption of dietary phosphate from the intestine exceeds daily requirements, thus necessitating mechanisms for removal to avoid toxicity. Vitamin D may play some role in the regulation of intestinal phosphate uptake, but likely only in circumstances of poor dietary phosphate content. Notably, studies in rodents suggest a much greater role for transcellular phosphate transport, suggesting caution in extrapolating rodent studies to human beings [15,16].

In the kidney, reabsorption of filtered phosphate occurs almost exclusively in the proximal tubule through the activity of three sodium-dependent phosphate cotransporters: Npt2a (type IIa sodium phosphate cotransporter; SLC34A1; NaPi IIa), Npt2c (type IIc sodium phosphate cotransporter; SLC34A3; NaPi IIc), and PiT2 (type III sodium phosphate cotransporter; SLC20A2; GLVR2; RAM1) [17]. The contribution of each of these transporters to phosphate homeostasis in humans remains an unanswered question. Most investigators in the field would agree that PiT2 plays a relatively minor role [18]. In rodents, where most of the basic studies have been performed, Npt2a accounts for 70–80% of filtered phosphate retrieval, while Npt2c has a lesser contribution. In fact, rodents in which Npt2c has been eliminated show no abnormalities of phosphate homeostasis [19,20]. The case is different for human beings, however. Mutations in the genes encoding either protein yield significant clinical sequelae, including low bone mass and kidney stones [3,21,22,23,24]. Genome-wide association studies, however, do identify loci in the region of Npt2a Pas significant determinants of serum phosphate levels [25].

Bone is the major repository for phosphate, accounting for 85% of the total body content [1]; there, it exists with calcium as a component of hydroxyapatite, the major form of bone mineral. Bone regulates serum phosphate through the sensing of serum phosphate levels and subsequent adjustment of the elaboration of the hormone FGF23 (fibroblast growth factor 23) [26,27,28,29,30,31]. However, bone phosphate uptake and release are not primary mechanisms for the control of serum phosphate. For example, in hyperparathyroid states, PTH will stimulate bone release of phosphate along with calcium, while parathyroidectomy or administration of high-dose bone resorption inhibitors, such as bisphosphonates or denosumab, will stimulate bone uptake of phosphate. These clinical scenarios infrequently result in changes in serum phosphate if intestinal and kidney function are intact.

Npt2a and Npt2c localize to the apical membrane of the kidney proximal tubule and do not require post-translational modifications for activation or inhibition [32,33,34,35,36]; therefore, the major mechanism for the regulation of proximal tubule transport of phosphorus is the trafficking of these proteins. Npt2a amino- and carboxy-terminal sequences are both intracellular and express multiple motifs for protein interactions that have documented roles in regulating their intracellular localization [36,37,38,39,40,41,42,43,44,45,46]. The cellular localization of Npt2a and Npt2c may also be mediated secondarily by proteins that interact with other trafficking proteins. This review will focus primarily on the proteins identified that regulate the trafficking of Npt2a and Npt2c. While several hormones upregulate (angiotensin II and IGF-1) or downregulate (dopamine, PTH, and FGF23) the apical membrane expression of these proteins, the majority of studies have focused on the two major hormonal regulators, PTH and FGF23 (reviewed in [47]).

## 3. Npt2a-Interacting Proteins

### 3.1. PDZ Proteins

The apical membrane expression of Npt2a is enhanced primarily by members of the family of PDZ (post-synaptic density protein PSD95, Drosophila disc large tumor suppressor Dig1, and zonula occludens-1 protein zo-1) proteins [42,48,49,50,51,52]. PDZ domains are amino acid sequences that, despite significant sequence dissimilarity, assume similar secondary structures [48,52,53]. The sequence lengths can be fairly long, with a globular structure that has six β strands, two α helices, and a solitary binding site within a hydrophobic region. These proteins generally bind distinct three-amino-acid motifs located on the carboxyterminal of a variety of proteins, including Npt2a, serving as scaffolds to link transporters, receptors, and other proteins into regulatory complexes. In kidney epithelial cells, these PDZ proteins play significant roles in the regulation of membrane protein expression, cell polarity, and intracellular signaling [54,55,56].

Six PDZ proteins have been identified as Npt2a-binding partners: NHERF (Sodium Hydrogen Exchanger Regulatory Factor) 1, 2, 3, and 4; Shank2E (SH3 and multiple ankyrin repeat domains protein 2E); and PIST (PDZ-domain protein interacting specifically with TC10). The NHERF family is characterized by the expression of more than one PDZ domain. Each family member has 3–4 names, a result of identification of their roles in diverse cell functions, and publications continue to use the alternative names (Table 1). The most extensively studied in kidney physiology is NHERF1 [57,58,59,60,61,62]. NHERF1 expresses two PDZ domains, each with specific, non-overlapping partners. An additional feature is an ezrin-binding domain, allowing NHERF1 to interact with ezrin, which, in turn, links NHERF1 to the apical membrane microtubule network. This protein complex, which also includes the PTH receptor and components of the PTH receptor signaling cascade, provides the framework for the compartmentalized PTH regulation of Npt2a apical membrane localization [63]. FGF23, the other major systemic hormonal regulator of Npt2a expression and function, acts through the FGFR1 receptor to activate Ras/ERK and SGK1 and ERK [64,65,66,67]. Activation of either PTH or FGF23 receptors causes phosphorylation of G protein-coupled receptor kinase 6a (GRK6A) that phosphorylates NHERF1, followed by dissociation of NHERF1 from Npt2a, and internalization of Npt2a into clathrin-coated vesicles [68,69,70,71,72]. These vesicles merge with early endosomes and eventually are shuttled to lysosomes for degradation. The actions of the two hormones are independent.

Two additional PDZ proteins, NHERF3 (PDZK1) and SHANK2E, also bind Npt2a at the apical membrane. NHERF3 binds more avidly to Npt2c and, thus, may play a more important role in the regulation of that transporter [73,74,75]. NHERF3 has four PDZ domains, enabling interactions with multiple proteins, including itself for homodimerization. Unlike NHERF1, NHERF3 does not express an ezrin-binding domain and, thus, may have a more prominent role in the intracellular trafficking of Npt2a; however, NHERF3 is also localized at the apical membrane of proximal tubule cells [43,74,76,77,78,79]. Like NHERF1, NHERF3 has been implicated in the regulation of multiple epithelial transporters and signaling proteins in kidney and other tissues [55,80,81,82,83]. SHANK2E expresses a single PDZ domain plus an ankyrin domain (ANK), a Src homology 3 domain (SH3), proline-rich regions (PRR), and a sterile alpha motif (SAM) domain. A recent paper also has identified a FERM (Band 4.1, Ezrin, Radixin, Moesin) domain at the amino terminus, suggesting that, like NHERF1, SHANK2E stabilizes Npt2a at the apical membrane through interaction with the microtubule network [84,85]. There are no studies examining a role for hormone regulation of the SHANK2E-Npt2a complex. However, in the presence of a high-phosphate stimulus, SHANK2E is internalized with Npt2a, shuttling Npt2a to the trans-Golgi network for eventual transport to the lysosome for degradation [86]. The roles for NHERF1 and SHANK2E in regulation of Npt2a expression and function diverge. NHERF1 appears to mediate hormone-mediated Npt2a internalization through dissociation, while SHANK2E mediates phosphate-stimulated Npt2a internalization as a unit. The SHANK family of proteins has been best studied in neurologic and psychiatric disorders where, predictably, based on their PDZ-binding domains, they participate in the trafficking and signaling of critical components of neurotransmission [85,87,88,89,90]. These proteins have also been studied in cardiovascular physiology [91]. The SHANK2E isoform has been described only in two tissues: liver and kidney [84,92].

PIST expresses only a single PDZ domain and appears to anchor Npt2a in the trans-Golgi network [48,93]. The protein also contains two coiled-coil regions, one with a leucine zipper motif [94]. Aside from the two referenced manuscripts, no further study of the role of PIST in the regulation of Npt2a expression has been undertaken. However, PIST has multiple demonstrated roles in the kidney and other tissues, including the regulation of tight junctions [95], trafficking of multiple proteins [75,96,97,98,99,100,101,102], and scaffolding of signaling molecules [94,103,104,105,106,107,108].

NHERF2 and NHERF4 binding to Npt2a have been demonstrated in in vitro studies. NHERF2 is highly homologous to NHERF1, including expression of two PDZ domains and an ezrin-binding domain. Its expression in proximal tubules is very low, and mice lacking NHERF2 expression do not show a phosphate-wasting phenotype, suggesting that it is unlikely to play a role in regulation of Npt2a expression or function [109]. However, NHERF1, NHERF2, and NHERF3 heterodimerize with each other, suggesting the possibility for a supportive role for NHERF2 in the regulation of Npt2a that has not yet been identified. NHERF4 shows high homology to NHERF3, including the expression of four PDZ domains and no ezrin-binding domain. Its greatest expression in proximal tubules is in subapical vesicles. NHERF4 interacts with MAP17 (discussed later) in the process of Npt2a internalization [110]. While the impact of these two members of the NHERF family on phosphate homeostasis and/or kidney phosphate transport remains unclear, very little investigation in this realm has been published. Their demonstrated interactions with the other members of the NHERF family and evidence of either direct or indirect interaction with Npt2a point toward a regulatory role, but the nature remains unclear [42,48,77,78,111,112].

The coordinated network of PDZ proteins that interact with Npt2a highlights the prominent role of the carboxyterminal PDZ-binding motif (-TRL) in the regulation of Npt2a trafficking. The studies suggest differing mechanisms for receptor-mediated vs ambient phosphorus-mediated mechanisms for retrieval of Npt2a from the apical membrane. PTH and FGF23 downregulate Npt2a expression through phosphorylation of NHERF1, inducing dissociation from Npt2a, which initiates Npt2a internalization. The internalized Npt2a is incorporated into a clathrin-coated vesicle, which then merges into early endosomes, eventually fusing with lysosomes for degradation. In contrast, in the presence of high extracellular phosphorus, SHANK2E—which also binds Npt2a at the apical membrane—is co-internalized with Npt2a, trafficked to the trans-Golgi network, and eventually degraded by lysosomes with Npt2a. The relative distribution of NHERF1-associated vs SHANK2E-associated Npt2a at the apical membrane has not been determined. Most studies suggest that NHERF1 binds only a fraction of apical membrane Npt2a but would be able to confer very rapid, tight, and flexible regulation of Npt2a expression in response to systemic hormonal stimuli in a concentration-dependent fashion. In addition, the persistent presence of NHERF1 at the apical membrane would ensure the ability of Npt2a to localize to the BBM in the event of an immediate need. It is possible that SHANK2E binds the remainder of the Npt2a expressed on the apical membrane, acting in response to local phosphate availability. SHANK2E-bound Npt2a may represent a mechanism by which the proximal tubule could sense and respond to high-filtered phosphate loads, particularly post-prandially, to limit reabsorption of excessive filtered phosphate. The turnover of SHANK2E is faster than NHERF1, consistent with its co-internalization and degradation with Npt2a, suggesting perhaps less of a regulatory role and more of a simple trafficking role [92]. How the hormonal- vs phosphate-mediated retrieval processes are integrated remains a mystery. Previous studies have demonstrated blunted PTH-induced phosphaturia in animals fed a low-phosphate diet, likely through alteration of cAMP signaling [113,114]. In humans, this phenomenon may be mediated by RGS14 binding to NHERF1 (discussed below) [115]; however, whether this, or a similar process, occurs in animals has not been determined.

### 3.2. Additional Npt2a-Interacting Proteins

#### 3.2.1. MAP17

Microtubule-associated protein 17 (MAP17) is an apical membrane protein with a single transmembrane domain that binds to the amino terminus of Npt2a [43]. The protein is expressed only at the brush border of the kidney proximal tubule in normal tissue, but is abundantly expressed in several tumor lines where the staining is predominantly in glandular structures [116,117]. In these cell culture models, the protein localizes to sites of cell–cell contact, suggesting a role for tight junction integrity [116], although this specific site of localization has not been described in the kidney. Most of the studies on this protein have focused on the interaction with SGLT2 (SLC5A2). MAP17 is required for SGLT2 function, and genetic analyses of individuals with congenital renal glycosuria have described pathogenic variants in MAP17 [118,119,120,121,122]. Heart failure is associated with increased SGLT2/MAP17 complexes in kidney and neural tissues, while the SGLT2 inhibitor empagliflozin enhances MAP17 expression in kidney, also emphasizing the important role for MAP17 in SGLT2 function. MAP17 appears to increase SGLT2 activity without increasing BBM SGLT2 protein expression. MAP17 interacts with a variety of transporters, including NHE3, Npt2a, and Npt2c [43,73,110,123,124], as well as PDZK1 (accounting for its alternative name, PDZK1-interacting protein [PDZK1IP1]) and possibly PDZK2 [77,117,124,125,126]. The interaction of MAP17 with PDZK1 is most likely mediated through the three C-terminal amino acids of MAP17, which form a PDZ-binding domain [43]. The role for MAP17 in the regulation of Npt2a is complex. MAP17 expression is not required for apical membrane Npt2a expression [110]. Overexpression of MAP17 results in diminished apical membrane expression of Npt2a due to sequestration in the trans-Golgi network via creation of a MAP17-PDZK1-Npt2a complex [43,110]. MAP17 also may mediate the internalization of Npt2a by dopamine, whether administered exogenously or produced endogenously by a high-phosphate diet [110]. The rather broad array of binding partners for MAP17 and its exclusive localization to the renal proximal tubule in healthy organisms suggest that it may mediate a common pathway in the regulation of the expression of these proximal tubule transporters, stimulated by various agonists. Alternatively, the protein may be a scaffold to link BBM transporters. This latter hypothesis is appealing, as recent studies have demonstrated an interdependence of transporter function. For example, Zhou et al. have reported that inhibition of SGLT2 transport blunted glycerol-3-phosphate production stimulated by Npt2a-mediated phosphate transport [127].

#### 3.2.2. TMEM174

In 2022, two manuscripts were published describing a role for TMEM174 (Transmembrane Protein 174) in the regulation of Npt2a BBM expression [128,129]. TMEM174, a member of a somewhat diverse family of proteins that express transmembrane domains, has two transmembrane domains and is expressed almost exclusively in the renal proximal tubule [130,131,132]. However, the protein is also expressed in multiple cancers, particularly kidney cancers, where it functions as an activator of cell proliferation and migration through stimulation of AP-1 transcription factor, CREB, and proliferative kinases such as cyclin D1 kinase and MAP kinase [130,131,132]. Using two different approaches, the authors of these two manuscripts identified the gene encoding TMEM174 as being closely related to SLC34A1 in the kidney proximal tubule [128,129]. TMEM174 is expressed in the apical membrane [129]. Knockdown of TMEM174 resulted in hyperphosphatemia; increased apical membrane expression of Npt2a; the failure of a high-phosphate diet to downregulate Npt2a; and inability of either PTH or FGF23 to downregulate Npt2a expression, despite preservation of the activation of the expected signaling pathways through their respective receptors. In fact, high dietary phosphate led to markedly exaggerated increases in serum phosphate, PTH, and FGF23 in TMEM174-deficient animals. The absence of TMEM174, however, did not prevent internalization of Npt2c, nor did it affect the expression of NHERF1 or PiT2 protein in the presence of a high-phosphate diet. TMEM174 knockdown animals also showed diminished gene expression of Npt2c and PiT2 under high-phosphate dietary conditions, consistent with a role for regulation of Npt2a alone. Levels of PTH and FGF23 were increased at the mRNA and protein levels [129]. Several questions remain in explaining how TMEM174 fits into the regulatory repertoire for Npt2a. TMEM174 was identified at the apical membrane of the proximal tubule. Interaction with NHERF1 was demonstrated in the presence but not the absence of Npt2a, suggesting that the interaction with NHERF1 was indirect through Npt2a. Somewhat paradoxically, a low-phosphate diet increased TMEM174 expression, while deletion of either Npt2a or Npt2c decreased TMEM174 expression [129]. Exogenous PTH also increased TMEM174 expression [128]. Levels of 1,25 dihydroxycholecalciferol were not different between wild-type and TMEM174-deficient animals. Levels of 1 alpha hydroxylase were similar, while levels of 24 hydroxylase were higher in the TMEM174-deficient animal kidneys [129]. The sustained hyperphosphatemia in the TMEM174-deficient animals was accompanied by vascular calcification, skeletal abnormalities, an enhanced susceptibility to acute kidney injury, and decreased longevity [120,129,133]. Overall, the studies suggest that TMEM174 is a critical and essential negative regulator of Npt2a expression; however, the mechanism remains unclear. The fact that two proteins with opposing effects on Npt2a function bind Npt2a at the apical membrane is both counterintuitive and perplexing. The observation that NHERF1 antibody immunoprecipitates TMEM174 in the presence but not the absence of Npt2a suggests that TMEM174 binds Npt2a but not NHERF1 directly, although this could also be consistent with a requirement for NHERF1-Npt2a binding. TMEM174 may enhance the ability of PTH or FGF23 to phosphorylate NHERF1—the step required for initiating internalization of Npt2a. TMEM174 may de-stabilize the Npt2a/NHERF1 complex by another mechanism, alter the conformation of Npt2a to decrease phosphate transport, or promote the interaction of Npt2a with another PDZ protein, such as PDZK1 or SHANK2E, involved in endocytosis or trans-Golgi network retention. A potential role for dysfunction or loss of TMEM174 in the development of hyperphosphatemic states, such as chronic kidney disease, has not been explored. The absence of TMEM174 provoked exaggerated PTH and FGF23 elevations, particularly in the setting of high-phosphate intake, which could suggest a role for diminished TMEM174 in the development of secondary hyperparathyroidism and FGF23 elevation in chronic kidney disease. Neither study addressed the possibility of direct effects of either PTH or FGF23 on TMEM174 expression, though the observation that Npt2a depletion decreased TMEM174 expression suggests that the two phosphaturic hormones that cause reduced Npt2a expression may have the same effect.

#### 3.2.3. RGS12 and 14

Regulator of G protein signaling isoforms 12 and 14 are members of a family of GTPase-accelerating proteins that terminate signaling pathways stimulated by G protein-coupled receptors [134]. The subfamily that includes RGS12 and 14 expresses the classic RGS domain, two Ras/Rap-binding domains, and a G protein regulatory domain (GPR or GoLoco) [135,136]. The classic RGS domain binds to activated Gα subunits, expresses GTPase activity and, thus, halts G-coupled hormone receptor signaling. The Ras/Rap-binding domains regulate Ras-MEK signaling by binding activated H-Ras and Rap2, influencing Raf kinases and calcium/calmodulin kinases. The GPR domain blocks Gαi GDP exchange and serves to scaffold RGS14 to the BBM. Human RGS12 and 14 express a C-terminal PDZ-binding motif, absent in rodents, allowing them to bind human NHERF1 [137,138]. The effect of the interaction between RGS12/14 and NHERF1 is to stabilize the NHERF1-Npt2a complex at the apical membrane. The outcome is to block both PTH- and FGF23-mediated Npt2a downregulation without downregulation of hormone receptor signaling [115]. The site on RGS14 that mediates this effect lies in a region between the RGS domain and the first Ras/Rap-binding domain [139]. The C-terminal PDZ-binding motif allows RGS12/14 to bind to NHERF1, creating an RGS12/14/NHERF1/Npt2a/ezrin complex in the apical membrane. Phosphorylation of residues within the serine-rich region between the RGS and RBD1 domains is another mechanism for regulating hormone-mediated phosphate uptake. RGS12 and 14 regulate the phosphaturic effect of the PTH receptor, which signals through a classic G protein-coupled receptor PTH1r, and the phosphaturic effect of FGF23, which signals through a tyrosine kinase receptor FGFR1 via noncanonical mechanisms, not involving the regulation of hormone signaling. These studies have identified novel mechanisms for RGS proteins to regulate hormone-mediated effects as well as novel binding partners for Npt2a. How the interaction of the RGS proteins with the NHERF family proteins is regulated remains unknown. Studies thus far have demonstrated interaction of RGS14 with NHERF1, but potential interactions with the other PDZ proteins involved in the regulation of Npt2a and Npt2c expression may also be enlightening. Human RGS14 variants have been identified and associated with serum phosphate, PTH, and FGF23 levels as well as kidney stones, chronic kidney disease, salt sensitivity, and even heart failure [140,141,142,143,144,145]. All of these associations may stem from abnormal phosphate homeostasis due to altered renal handling, making RGS14 a tempting therapeutic target.

The species difference in the expression of a PDZ-binding domain in human but not rodent RGS14 is critical for the translation of the studies of renal phosphate handling, which have been performed primarily in rodents. This extra layer of regulation of phosphate handling in the human kidney likely accounts for the observations that variants in the region of RGS14 are associated with multiple clinical mineral metabolism parameters. As serum phosphate levels are a major therapeutic target, this interspecies difference in the properties of RGS14 suggests that further mechanistic studies of the role of serum phosphate in cardiovascular risk will need to be performed in humans, animal models that express RGS14 with the PDZ-binding domain, or animal models that express the humanized RGS14.

#### 3.2.4. PEX19

PEX19 is a protein involved in the formation and insertion of membrane proteins in peroxisomes [146]. The protein expresses a PEX3 interaction domain and a CAAX myristoylation domain that is required for its membrane association. While initially thought to be specific to peroxisomes, evidence suggests that PEX19 may also be involved in the chaperoning and insertion of proteins into the membranes of lipid droplets, mitochondria, and other organelles [147,148,149]. PEX19 binds to a KR motif on one of the intracellular loops of Npt2a in response to PTH activation of its receptor, leading to internalization [150]. Mutation of this motif abolished PTH action. Overexpression of PEX19 stimulated internalization of Npt2a in the absence of hormonal stimulation. PEX19, therefore, appears to be an essential component of PTH-mediated Npt2a internalization, likely through its association with the cell machinery involved in clathrin-mediated endocytosis—the mechanism by which Npt2a is internalized [151,152,153,154]. Whether PEX19 is also required for Npt2a internalization mediated by other regulators of Npt2a expression, such as FGF23, dopamine, or high phosphate, is not known; however, PEX19 expression does increase in the presence of PTH and high phosphate [150]. Interestingly, PEX19 binds to NHERF1 and NHERF3 [150]. Whether this interaction is important for the membrane localization of these proteins or whether the interaction serves as a negative regulator of NHERF1-mediated apical membrane expression of Npt2a is unknown.

Peroxisome abnormalities have been implicated in kidney conditions, including primary hyperoxaluria, cystic kidney diseases, diabetic kidney disease, and acute kidney injury to chronic kidney disease transition (reviewed in [148,155,156,157,158]). However, a role for a peroxisomal protein in the regulation of a kidney transport protein is unique. Although not yet proven, this mechanism for internalization of Npt2a may also be applicable to FGF23, dopamine, and even high-phosphate-mediated downregulation of Npt2a expression. Potential roles for PEX19 could be in the transport of Npt2a between the apical membrane and clathrin-coated vesicles, early endosomes, or the trans-Golgi network.

#### 3.2.5. Vilip3

Neural visinin-like proteins are a family of calcium-sensor proteins that express multiple EF-hand motifs [159,160]; they have been studied primarily in neural tissue and are implicated in the development of central nervous system disorders such as Parkinsonism. The proteins are characterized by the expression of four EF-hand motifs and a calcium-dependent myristoylation site that enables membrane association. Vilip3 (also known as NVP-3 or REM-1) has been identified in both proximal and distal nephron, the latter being more prominent [161]. A potential association with the Npt2a N-terminus was suggested by yeast two-hybrid studies, and immunohistochemistry identified the protein at the apical membrane of the kidney proximal tubule; however, in situ co-association has not been confirmed. The role of this protein in the regulation of Npt2a has not been further studied. However, documented roles for the Vilip proteins in calcium sensing and membrane trafficking suggest that further study in the kidney may be fruitful. Proximal tubule cells do express the calcium-sensing receptor and mediators of Npt2a trafficking, such as PTH-mediated calcium signaling mechanisms, justifying continued interest in this protein [162,163,164].

#### 3.2.6. GABARAP

The GABA-A Receptor-Associated Protein (GABARAP) is a member of the ATG8 family of proteins involved in autophagy, where GABARAP plays a major role in the late stages, facilitating fusion of autophagosomes to lysosomes for degradation [165,166,167]. Prior studies had identified an upregulation of Npt2a in the absence of GABARAP [45,168]; however, no further study of a potential role for GABARAP in the regulation of Npt2a has been completed. These initial observations, however, suggest that GABARAP may play a role in either constitutive or hormone-/diet-mediated turnover of Npt2a. GABARAP is also a marker of autophagy. A recent study has suggested that Sirtuin 5 may regulate ammoniagenesis in non-liver organs, including the kidney, and that Sirtuin 5 deletion increased the expression of autophagy markers, including GABARAP [169]. Ammoniagenesis and the regulation of phosphate transport are key functions of the proximal tubule in acid–base balance [170]. These observations suggest a potential role for GABARAP in the regulation of Npt2a in the setting of metabolic acidosis or other conditions of increased ammoniagenesis.

## 4. Npt2c-Interacting Proteins

The investigation of Npt2c-interacting proteins is not as robust as for Npt2a; however, the recognition that mutations in the gene encoding Npt2c are responsible for the human disease, Hereditary Hypophosphatemic Rickets with Hypercalciuria, has accelerated interest in elucidating the regulation of this transporter [21,23,171,172,173,174,175,176,177,178,179,180]. In the process, exciting differences in the contribution of specific interactive proteins to the regulation of Npt2c when contrasted with Npt2a have emerged. Table 1 depicts significant differences between the properties of Npt2a and Npt2c, while Table 2 compares what is known about interacting proteins. Differences in transport properties, including electrogenicity and responses to hormones and dietary phosphate, have been well-documented [47,65,179,180,181,182]. Additional observations also highlight differences between the two proteins that likely play a role in their unique contributions to phosphate homeostasis. For example, Npt2a exists as a phosphorylated protein [35]. Mutation of the putative phosphorylation sites does not affect either baseline expression or hormone regulation [34]. In contrast, mutation of S138 on Npt2c decreases expression and response to PTH [32]. Potassium deficiency alters the lipid content of the apical membrane, leading to increased expression of Npt2a but decreased transport function. However, the expression of Npt2c is decreased [183,184,185]. Both effects of potassium deficiency likely contribute to phosphate wasting but through differing mechanisms. Pertinent to this review, the sequence differences in the C-terminal of Npt2a and Npt2 contribute to differential expression and regulation. While the apical membrane expression of Npt2a is highly dependent on interaction with NHERF1 through its C-terminal PDZ-binding motif (-TRL), Npt2c lacks a similar motif. However, both proteins bind NHERF1, which is required for the apical membrane expression of both. The PDZ-binding motif of Npt2c is embedded within the C-terminal tail, a five-amino-acid sequence WLHSL [186]. Interestingly, Sneddon and coworkers had also determined that an additional PDZ-binding motif existed within the C-terminal tail of Npt2a that, when deleted, did not block Npt2a expression but did block the response to PTH [187]. The authors proposed that the internal PDZ-binding motif bound to the second regulatory PDZ domain of NHERF1, instead of the usual binding to the first PDZ domain, as a potential mechanism.

Both Npt2a and Npt2c interact with NHERF1, NHERF3, and MAP17; however, investigators were unable to demonstrate an interaction between Npt2c and any of the other PDZ proteins [73]. The NHERF1 and NHERF3 interactions occur at the apical membrane, similarly to what is seen with Npt2a. Likewise, the interaction between Npt2c, NHERF3, and MAP17 regulates the translocation of Npt2c into the trans-Golgi network, while NHERF1 is not able to induce this translocation.

## 5. Conclusions

**Figure 1:**
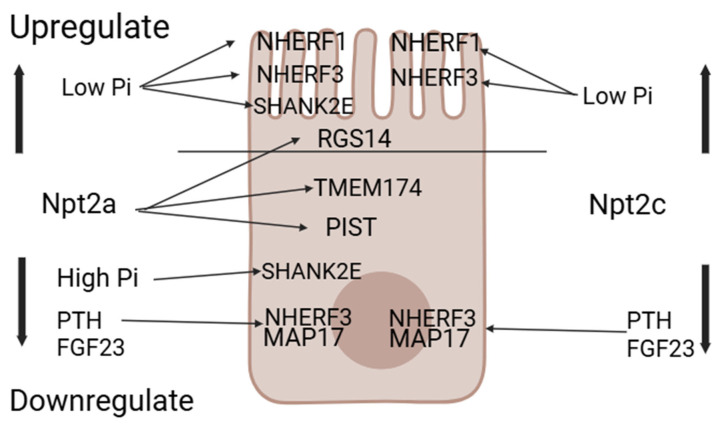
Comparison of factors that upregulate and downregulate Npt2a and Npt2c. This figure shows the Npt2a- and Npt2c-interacting proteins with documented effects on apical membrane localization. The left half of the figure is devoted to Npt2a, while the right half is devoted to Npt2c. Stimuli and interacting proteins above the horizontal line represent those associated with apical membrane upregulation, while stimuli and interacting proteins below the horizontal line represent those associated with decreased apical membrane expression. TMEM174 and PIST decrease apical membrane expression of Npt2a, but the stimuli are unknown. RGS14 stabilizes apical membrane expression of Npt2a.

Clearly, there is a wealth of interacting proteins that regulate the expression and, therefore, the function of Npt2a and Npt2c. The mechanisms by which these interacting proteins are globally regulated to eventuate in the final levels of Npt2a and Npt2c expression are not understood (Figure 1). While the data suggest that some of the interacting proteins may play a more dominant role, no single protein stands out as the major mediator of apical membrane expression of either transporter. Understanding of these mechanisms could have significant translational applications. Clinical syndromes of excessive phosphate retention, such as chronic kidney disease, hypoparathyroidism, and tumoral calcinosis, could be ameliorated by activation of proteins that block Npt2a apical membrane expression, such as TMEM174 and PIST, or inhibition of proteins that retain Npt2a apical expression, such as NHERF1, thus preventing the harmful effects of hyperphosphatemia on vascular and other soft tissue mineralization. Conversely, individuals with clinical syndromes associated with phosphate wasting, such as Fanconi syndrome or hypophosphatemic rickets, may benefit from augmentation of proteins that promote Npt2a apical membrane expression, such as NHERF1, or the inhibition of proteins that promote endocytosis, such as the combination of MAP17 and NHERF3. More investigation to define the differential contributions of the kidney phosphate transporters Npt2a, Npt2c, and PiT2 is needed to enable more precise targeting of the different phenotypes of aberrant phosphate homeostasis. Additional studies are needed to determine if, for example, overexpression of one of the proteins can compensate for failure of another or vice versa. Recent publications describing abnormal phosphate homeostasis even in the setting of heterozygous mutations of Npt2c would suggest that perhaps such compensatory responses do not occur [22,188]. The recent development of specific Npt2a inhibitors is an exciting step forward in ameliorating hyperphosphatemic syndromes [189]; however, these inhibitors have been tested in mice, not humans. The recognition that both Npt2a and Npt2c expression affect phosphate homeostasis highlights the need to study human phosphate homeostasis. The rapid pace of new knowledge acquisition in this field predicts newer and more targeted therapies are on the horizon.

Review of these studies also highlights the fact that while our understanding of the molecular biology of Npt2a expression and function has largely been drawn from studies in rodents, there are clear differences between rodent and human kidneys and intestinal handling of the SCL34 family of phosphate transporters. While rodents rely heavily on intestinal Npt2b for phosphate absorption, human intestinal phosphate transport is driven by uptake through paracellular pathways. Phosphate reabsorption from glomerular ultrafiltrate in the proximal tubule is almost exclusively through Npt2a in rodents. In contrast, in humans, mutations in either Npt2a or Npt2c cause significant clinical disease, indicating a far greater role for Npt2c in human phosphate homeostasis. These observations do not dismiss the contributions made in animal models, which have enabled us to identify and study mechanisms of phosphate transport at the molecular level. They do, however, remind us that, ultimately, if we wish to impact human phosphate homeostasis, we will need to study humans.

## Figures and Tables

**Table 1:** Comparison of Npt2a and Npt2c properties.

	Npt2a	Npt2c
Size (AA)	Mouse: 637, Human: 639	Mouse: 601, Human: 599
Localization	S1, S2, S3	S1
Transport Properties Electrogenicity pH Dependence Km Pi Km Na Pi Valence Preference	Electrogenic 3:1 Na:Pi Strong 50 uM 50 uM Divalent	Non-electrogenic 2:1 Na:Pi Strong 70–80 uM 50 uM Divalent
Phosphorylation Status	Multiple putative phosphorylation sites, none required for regulation of expression	Multiple putative phosphorylation sites, S138 important for expression and function
PTH Regulation	Rapid downregulation (1 h)	Slow downregulation (4 h)
High Dietary Pi Regulation	Rapid downregulation (1 h)	Slow downregulation (4 h)
Low Dietary Pi Regulation	Upregulation	Upregulation
Low Dietary K Regulation	Upregulation	Downregulation
PDZ-Binding Domain	C-terminal -TRL (all PDZ proteins)	C-terminal -QQL for NHERF3 Unknown for other PDZ proteins
PDZ Protein Interactions NHERF1 NHERF2 NHERF3 NHERF4 SHANK2E PIST	+BBM, −TGN + +BBM, +TGN + +BBM, +TGN +TGN	+BBM, −TGN +/−(conflicting results) +BBM, +TGN - - -
TMEM174 Protein Interaction	+subapical	-
RGS12/14 Protein Interaction	+indirect through NHERF1, human only	unknown
MAP17 Protein Interaction	+BBM, +/−NHERF1, with NHERF3 TGN	+BBM, −/+NHERF1, with NHERF3 TGN
Pex19	+	unknown
GABARAP	+likely indirect	unknown
Vilip 3	+likely indirect	unknown

+ documented interaction at indicated site; − no interaction at indicated site; +/− means conflicting results.

**Table 2:** Comparison of Npt2a- and Npt2c-interacting proteins and functional effects.

	NHERF1	NHERF2	NHERF3	NHERF4	SHANK2E	PIST	TMEM174	RGS12/14	MAP17
Primary Name in Context of Phosphate Transport	Na-H Exchanger Regulatory Factor 1	Na-H Exchanger Regulatory Factor 2	Na-H Exchanger Regulatory Factor 3	Na-H Exchanger Regulatory Factor 4	SH3 and multiple repeat domains protein, Isoform 2E	PDZ-domain protein interacting specifically with Tc10	Membrane-associated protein interacting with Slc34a1	Regulator of G Protein Signaling 14	PDZK1-interacting protein 1
Alternate Names	EBP-50 NPHLOP2 NHERF	E3KARP SIP-1 TAK-1 OCTS2	PDZK1 CAP70 NaPi-Cap1 PDZD1	DIPHOR-2 IKEPP NaPi-Cap2 PDZK2	ProSAP1 CortBP1 Spank-3 CTTNBP1	CAL Gopc FIG	MGC13034 FLJ31268	RGS12 family PDZ domain containing	MAP17 DD96SPAP 17KDa Membrane-Associated Protein
Genetic Alias	SLC9A3R1	SLC9A3R2	SLC9A3R3	SLC9A3R4	SHANK2E	GOPC1	TMEM174	RGS14	PDZK1IP1
Association and Functional Effect on NpT2a and Npt2c.	Directly associates with and regulates trafficking of NpT2a and Npt2c.	Cell membrane and nuclear scaffold (PMID: 18829453). Possible association with NpT2a, not Npt2c.	Scaffold protein at apical membrane for NpT2a and NpT2c. With MAP17, shuttle to TGN.	Scaffold protein at apical membrane for NpT2a, not Npt2c. With MAP17, shuttle to TGN.	Apical membrane binding of NpT2a, not Npt2c. Involved in phosphate-induced downregulation of NpT2a.	Binds Npt2a in Golgi. Does not interact with Npt2c.	Binds to NHERF1 in presence of NpT2a. Deletion leads to increased Npt2a and decreased NpT2c expression.	Binds to NpT2a likely through Slc9a3R1. Stabilizes Npt2a at apical membrane.	Binds Npt2a and Npt2c at N-terminal. Facilitates TGN transfer of internalized proteins.

## Data Availability

No new data were created or analyzed in this study. Not applicable.
